# Upregulation of MAPKAPK5‐AS1, PXN‐AS1 and URB1‐AS1 lncRNAs in non‐functioning pituitary adenoma

**DOI:** 10.1111/jcmm.17763

**Published:** 2023-05-08

**Authors:** Mohammad Taheri, Arash Safarzadeh, Arefe Bahranian, Solat Eslami, Nader Akbari Dilmaghani, Soudeh Ghafouri‐Fard, Guive Sharifi

**Affiliations:** ^1^ Institute of Human Genetics Jena University Hospital Jena Germany; ^2^ Urology and Nephrology Research Center Shahid Beheshti University of Medical Sciences Tehran Iran; ^3^ Phytochemistry Research Center Shahid Beheshti University of Medical Sciences Tehran Iran; ^4^ Skull Base Research Center, Loghman Hakim Hospital Shahid Beheshti University of Medical Sciences Tehran Iran; ^5^ Department of Medical Biotechnology, School of Medicine Alborz University of Medical Sciences Karaj Iran; ^6^ Dietary Supplements and Probiotic Research Center Alborz University of Medical Sciences Karaj Iran; ^7^ Men's Health and Reproductive Health Research Center Shahid Beheshti University of Medical Sciences Tehran Iran

**Keywords:** lncRNA, MAPKAPK5‐AS1, non‐functioning pituitary adenoma, PXN‐AS1, URB1‐AS1

## Abstract

Long non‐coding RNAs (lncRNAs) have been shown to be dysregulated in a variety of malignant and non‐malignant lesions including non‐functioning pituitary adenomas (NFPAs). In the current experimental study, we have selected six lncRNAs, namely MAPKAPK5‐AS1, NUTM2B‐AS1, ST7‐AS1, LIFR‐AS1, PXN‐AS1 and URB1‐AS1 to assess their expression in a cohort of Iranian patients with NFPA. MAPKAPK5‐AS1, PXN‐AS1 and URB1‐AS1 were shown to be over‐expressed in NFPA tissues compared with control samples (Expression ratios (95% CI) = 10 (3.94–25.36), 11.22 (4.3–28.8) and 9.33 (4.12–21.12); *p* values < 0.0001, respectively). The depicted ROC curves showed the AUC values of 0.73, 0.80 and 0.73 for MAPKAPK5‐AS1, PXN‐AS1 and URB1‐AS1, respectively. Relative expression level of PXN‐AS1 was associated with tumour subtype (*p* value = 0.49). Besides, relative expression levels of MAPKAPK5‐AS1 and LIFR‐AS1 were associated with gender of patients (*p* values = 0.043 and 0.01, respectively). Cumulatively, the current study indicates the possible role of MAPKAPK5‐AS1, PXN‐AS1 and URB1‐AS1 lncRNAs in the pathogenesis of NFPAs.

## 1 INTRODUCTION

Pituitary adenomas (PA) include a wide variety of anterior pituitary masses. Based on the statistics, clinically important pituitary neoplasms have an incidence of 80–100 per 100,000 persons.^1^ In a broad classification which is based on the levels of hormone release, pituitary neoplasms are classified as functional and non‐functioning pituitary adenomas (NFPAs). The latter type includes about one third of all PAs.^2^ Most of these tumours are considered as histologically benign tumours; however, the associated comorbidity and mortality of these tumours make them clinically important.^3^ The process of development of NFPA is dependent on numerous molecular events and biomolecules, among them being long non‐coding RNAs (lncRNAs).^4^, ^5^ These transcripts control expression of their targets at almost all supposed stages. Notably, malfunction of these transcripts has been shown to speed up the carcinogenesis process in several tumours, including PA.^6^, ^7^, ^8^, ^9^

Recently, we have used an in silico method to find differentially expressed long non‐coding RNAs (lncRNAs) in PAs versus normal samples and identify their relation with important signalling pathways.^10^ In the current experimental study, we have selected six lncRNAs from mostly upregulated lncRNAs namely, MAPKAPK5‐AS1 (Log FC = 1.360005, adjusted *p* value = 0.01), NUTM2B‐AS1 (Log FC = −2.536098, adjusted *p* value = 0.01), ST7‐AS1 (Log FC = 2.60673, adjusted *p* value = 0.04), LIFR‐AS1 (Log FC = 4.262626, adjusted *p* value = 0.007), PXN‐AS1 (Log FC = 4.322818, adjusted *p* value = 0.008) and URB1‐AS1 (Log FC = 3.614134, adjusted *p* value = 0.01) among differentially expressed lncRNAs to assess their expression in a cohort of Iranian patients with NFPA.

## 2 MATERIALS AND METHODS

### 2.1 Patients

Expression of mentioned lncRNAs was assessed in NFPA samples and paired normal samples. Tissue samples were excised during tumour excision from patients admitted to hospital affiliated to Shahid Beheshti University of Medical Sciences during 2021–2022. None of patients had received any chemo/radiotherapy before surgery. The study protocol was approved by the ethical committee of Shahid Beheshti University of Medical Sciences. Informed consent forms were obtained from all participants.

### 2.2 Expression assays

Total RNA was extracted using RNJia extraction kit (RN983006, Roje Technologies Company, Iran). Afterward, cDNA was made from these samples using AddScript cDNA Synthesis Kit (Cat. No. 22701, ADDBIO Company, South Korea). Expression of lncRNAs was determined using RealQ Plus 2× Master Mix Green with high ROX purchased (AMPLIQON, Denmark), and primers provided by the METABION Company (Germany). Table 1 shows information about primers.

**TABLE 1:** Information about primers and the corresponding amplified region.

Name	Type	Sequence	Primer length	PCR product length
URB1‐AS1‐F	lncRNA	CCGCAAACTGATGGGCTCTT	20	153
URB1‐AS1‐R		GGACTGGATAGGGTCGGCTT	20	
PXN‐AS1‐F	lncRNA	TAAGCACAAGACCTGGACACCT	22	139
PXN‐AS1‐R		CAAGGGCACAGTTGAGGATGG	21	
NUTM2B‐AS1‐F	lncRNA	CGCTGGCTGAAGAGTTGATGAC	22	84
NUTM2B‐AS1‐R		AGTGAGGCGGAGAACACAGAG	21	
MAPKAPK5‐AS1‐F	lncRNA	CACAAATCACACTCACCAGGGAA	23	189
MAPKAPK5‐AS1‐R		TGGCGTTCTTCTCGGCTCT	19	
LIFR‐AS1‐F	lncRNA	TGGGTCTCTAAACAAGGGCTG	21	168
LIFR‐AS1‐R		GCCTCCATTTTGTGAAGGTGT	21	
ST7‐AS1‐F	lncRNA	TCCCTACAAGTGGCTTTCGT	20	128
ST7‐AS1‐R		CTGAGGCGTTTCCTTCGG	18	
B2M‐F	mRNA	AGATGAGTATGCCTGCCGTG	20	105
B2M‐R		GCGGCATCTTCAAACCTCCA	20	

### 2.3 Statistical analysis

Microarray data were processed using the R statistical programming language as described previously.^10^ Batch effects were removed by applying the ComBat function from the R Package Surrogate Variable Analysis (SVA). Subsequently, quantile normalisation method was used to normalize data expression matrix. Quantile normalisation was performed using the preprocessCore R package. The Limma package in R language was used to identify differentially expressed lncRNAs between NFPA and normal samples.

SPSS v.22.0 (SPSS Inc., Chicago, IL) was used for analyses. Graphics were created using GraphPad Prism version 9.0 (GraphPad Software, La Jolla California USA). Expression levels of six lncRNAs, namely MAPKAPK5‐AS1, NUTM2B‐AS1, ST7‐AS1, LIFR‐AS1, PXN‐AS1 and URB1‐AS1 genes were compared between in pituitary adenoma samples and adjacent normal tissues. Expression levels in each sample were calculated using the efficiency‐adjusted Ct of normalizer gene (B2M)—efficiency‐adjusted Ct of target gene method. The normal/Gaussian distribution of the values was assessed by the Shapiro–Wilk test. Wilcoxon matched‐pairs signed rank test or paired *t* test was used to identify differentially expressed genes between the adenoma tumour tissues and adjacent normal tissues.

Correlation between expressions of studied genes was measured using Spearman correlation coefficient. Mann–Whitney test and Kruskal–Wallis one‐way anova were used for comparing gene expression levels between different subgroups of patients. Chi‐square test was used to find out the association between clinicopathological factors and gene expression levels.

The receiver operating characteristic (ROC) curve was illustrated by the GraphPad Prism v.9 software. The *p* value < 0.05 was considered to define the statistical significance in all of measurements.

## 3 RESULTS

### 3.1 General information about studied genes

Table 2 shows the information about the studied genes.

**TABLE 2:** Characteristic features of genes studied in this article.

Name/Gene ID	Accession number	Location	Official full name	Gene type
MAPKAPK5‐AS1	NR_015404.2; NR_152605.1; NR_152606.1; NR_152607.1; NR_152608.1	12q24.12	MAPKAPK5 antisense RNA 1	ncRNA
NUTM2B‐AS1	NR_120611.1; NR_120612.1; NR_120613.1	10q22.3	NUTM2B antisense RNA 1	ncRNA
ST7‐AS1	NR_002330.1	7q31.2	ST7 antisense RNA 1	ncRNA
LIFR‐AS1	NR_103553.1; NR_103554.1	5p13.1	LIFR antisense RNA 1	ncRNA
PXN‐AS1	NR_038924.1	12q24.23	PXN antisense RNA 1	ncRNA
URB1‐AS1	NR_026845.1	21q22.11	URB1 antisense RNA 1	ncRNA

### 3.2 General information about enrolled persons.

Table 3 shows general information about enrolled patients. More details are shown in Table S1.

**TABLE 3:** General information about enrolled persons.

Parameters	Values
Gender (male/Female)	35/12
Age (Mean ± standard deviation)	50.65 ± 12.46
Tumour size	Macro: 34
	Giant: 7
	Micro: 2

### 3.3 Expression assays

**FIGURE 1:**
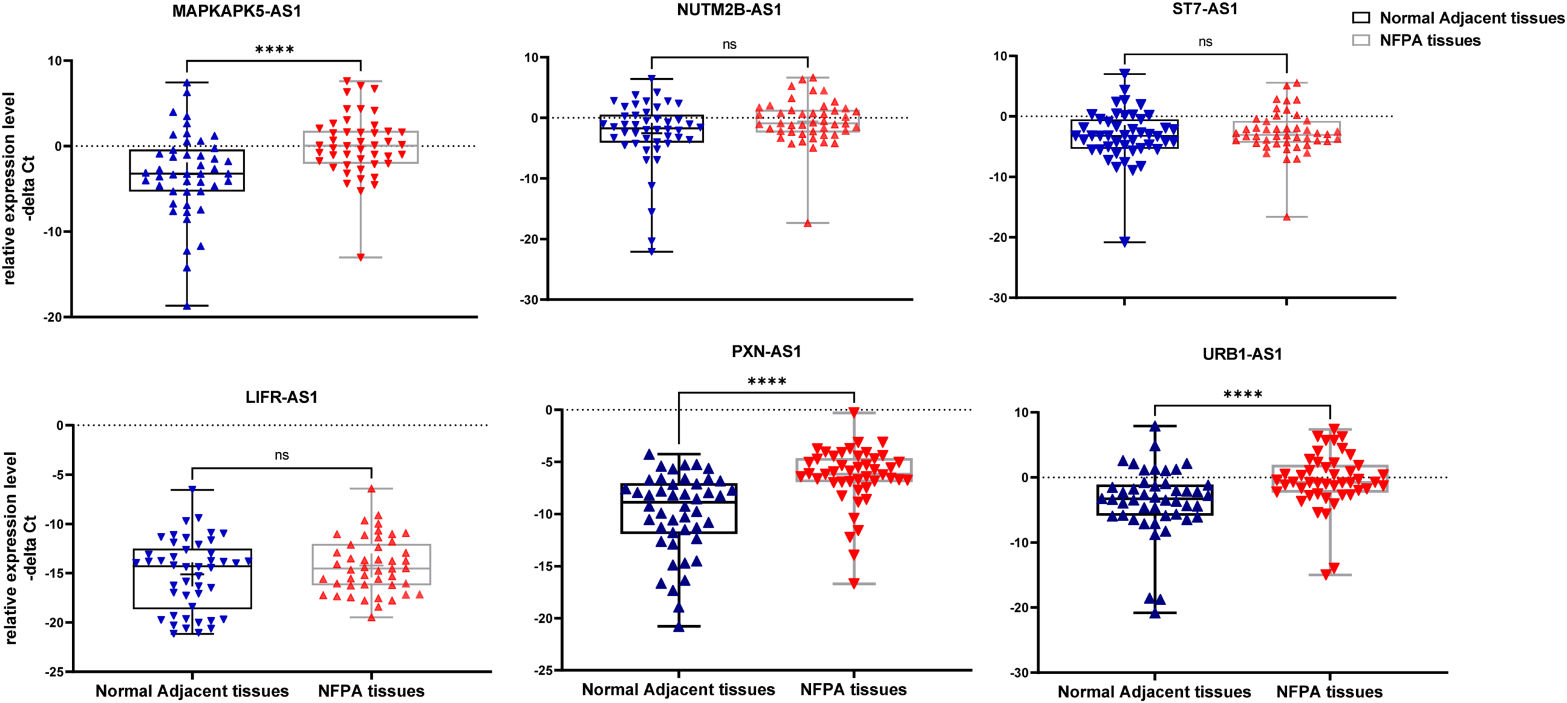
Relative expression levels of six lncRNA genes in non‐functional pituitary adenoma (NFPA) tissues versus adjacent normal tissues as described by—delta Ct values (Ct Housekeeping gene‐ Ct Target gene)—delta Ct Data were plotted as box and whisker plots Median [line], mean [cross], interquartile range [box], and minimum and maximum values are shown. Data were analysed using the Wilcoxon rank‐sum test or paired *t* test, and *p* < 0.05 was considered as statistically significant. Asterisks indicate significant difference between two mentioned groups (*p value < 0.05, **p value < 0.01 ns; non‐significant).

Expression levels of MAPKAPK5‐AS1, PXN‐AS1 and URB1‐AS1 were significantly different between NFPA samples and their corresponding non‐cancerous tissues (Figure 1).

MAPKAPK5‐AS1, PXN‐AS1 and URB1‐AS1 were shown to be over‐expressed in NFPA tissues compared with control samples (Expression ratios (95% CI) = 10 (3.94–25.36), 11.22 (4.3–28.8) and 9.33 (4.12–21.12); *p* values < 0.0001, respectively). Table 4 shows the detailed statistics of expression assays.

**TABLE 4:** The results of expression study of six lncRNA genes in non‐functional pituitary adenoma (NFPA) tissues compared with the adjacent normal tissues.

Studied genes	Expression ratio (95% CI)	Number of pairs	SEM	*p* Value
MAPKAPK5‐AS1	10 (3.94–25.36)	46	0.66	<0.0001
NUTM2B‐AS1	3.85 (1.07–13.86)	46	0.91	0.08
ST7‐AS1	1.45 (0.6–3.5)	46	0.63	0.54
LIFR‐AS1	1.8 (0.8–4.3)	46	0.6	0.14
PXN‐AS1	11.22 (4.3–28.8)	46	0.67	<0.0001
URB1‐AS1	9.33 (4.12–21.12)	46	0.58	<0.0001

*Note*: The expression ratio of each gene is shown as mean and 95% Confidence interval and SEM.

**FIGURE 2:**
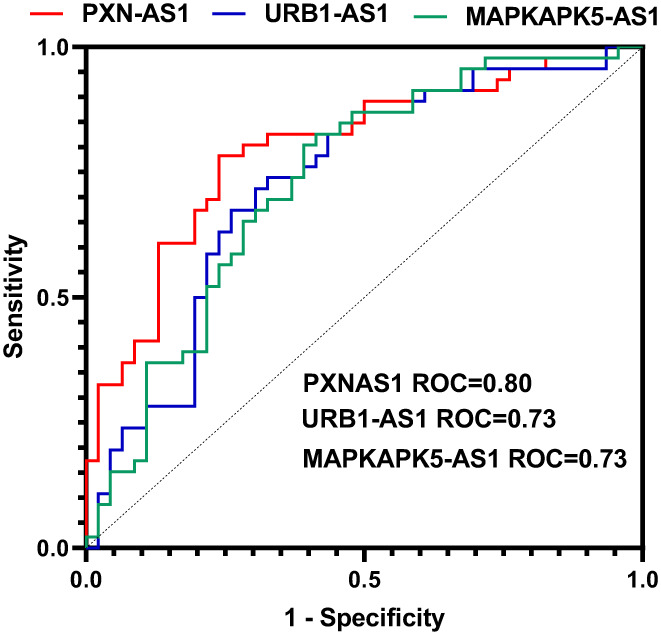
The receiver operating characteristic (ROC) curve of PXN‐AS1, URB1‐AS1 and MAPKAPK5‐AS1 lncRNA genes for discrimination of NFPA tumours from adjacent normal tissues. AUC indicates area under the ROC curve.

The depicted ROC curves showed the AUC values of 0.73, 0.80 and 0.73 for MAPKAPK5‐AS1, PXN‐AS1 and URB1‐AS1, respectively (Figure 2).

The highest sensitivity value belonged to MAPKAPK5‐AS1 (0.83), and the highest specificity value belonged to PXN‐AS1 (0.76). Table 5 shows detailed information about ROC curve analyses.

**TABLE 5:** The results of ROC curve analysis for six LncRNA genes for discrimination of NFPA tumours genes from adjacent normal tissues.

PXN‐AS1				URB1‐AS1				MAPKAPK5‐AS1			
AUC ± SD	Sensitivity	Specificity	*p* Value	AUC ± SD	Sensitivity	Specificity	*p* Value	AUC ± SD	Sensitivity	Specificity	*p* Value
0.8 ± 0.04	0.78	0.76	<0.0001	0.73 ± 0.05	0.72	0.69	0.0002	0.73 ± 0.05	0.83	0.58	0.0001

Correlation analyses revealed highest correlations between NUTM2B‐AS1 and MAPKAPK5‐AS1 in NFPAs (correlation coefficient = 0.95) and between ST7‐AS1 and MAPKAPK5‐AS1 among control tissues (correlation coefficient = 0.89). Table 6 shows the correlation coefficients for all lncRNA pairs in two sets of samples.

**TABLE 6:** Spearman's correlations between six lncRNA genes expression levels among the non‐functional pituitary adenoma tumour tissues (*N* = 46) and adjacent normal tissues (*N* = 46).

	NUTM2B‐AS1		ST7‐AS1		LIFR‐AS1		PXN‐AS1		URB1‐AS1	
	NFPAs	Control	NFPAs	Control	NFPAs	Control	NFPAs	Control	NFPAs	Control
MAPKAPK5‐AS1	0.95**	0.64**	0.8**	0.89**	0.006	0.36*	−0.04	0.26	0.72**	0.70**
NUTM2B‐AS1			0.85**	0.72**	0.16	0.23	−0.01	0.22	0.73**	0.48**
ST7‐AS1					0.33	0.48**	0.05	0.37*	0.78**	0.78**
LIFR‐AS1							0.17	0.40*	0.17	0.43*
PXN‐AS1									0.08	0.41*

* Significance level of *p* < 0.05.

** Significance level of *p* < 0.001.

There was a significant positive association between age of NFPA patients and invasiveness of NFPA (χ^2^ = 4.24, *p* value = 0.039). Moreover, there was a significant positive association between diseases duration and CSF leak (χ^2^ = 11.4, *p* value = 0.023). Finally, there was a significant positive association between tumour size and Knosp classification (χ^2^ = 11.5, *p* value = 0.02) and invasiveness of NFPA (χ^2^ = 6.12, *p* value = 0.04).

Relative expression level of PXN‐AS1 was associated with tumour subtype (*p* value = 0.49). Besides, relative expression levels of MAPKAPK5‐AS1 and LIFR‐AS1 were associated with gender of patients (*p* values = 0.043 and 0.01, respectively). Table 7 summarizes these results.

**TABLE 7:** Comparison of expression levels of six lncRNA genes in NFPA samples with different clinicopathologic factors.

Parameters	Subclasses	Number of patients (%)	Relative expression level of MAPKAPK5‐AS1 (mean ± SD)	*p*‐Value	Relative expression level of NUTM2B‐AS1 (mean ± SD)	*p*‐Value	Relative expression level of ST7‐AS1 (mean ± SD)	*p*‐Value	Relative expression level of LIFR‐AS1 (mean ± SD)	*p*‐Value	Relative expression level of PXN‐AS1 (mean ± SD)	*p*‐Value	Relative expression level of URB1‐AS1 (mean ± SD)	*p*‐Value
Tumour subtypes	NFPA	33	−1.2 ± 1.96	0.71	−0.89 ± 1.62	0.75	−3.3 ± 1.62	0.81	−14.7 ± 1	0.96	−8.69 ± 1.5	**0.049**	−1.5 ± 2.2	0.9
	NFPA + CD + AP	11	−0.04 ± 0.68		−0.92 ± 0.7		−2.8 ± 0.65		−14.2 ± 0.49		−5.9 ± 0.46		−0.3 ± 0.7	
Age	22–48	22	0.31 ± 1	0.06	−0.26 ± 0.82	0.07	−3.2 ± 1	0.27	−14.8 ± 0.6	0.49	−6.6 ± 0.99	0.34	−0.9 ± 1.3	0.32
	49–77	22	−0.97 ± 0.92		−1.5 ± 1		−2.8 ± 0.72		−13.9 ± 0.66		−6.6 ± 0.44		−0.2 ± 0.7	
Gender	Female	11	−2.4 ± 1.3	**0.043**	−3.03 ± 1.56	0.096	−3.35 ± 0.65	0.57	−12.5 ± 0.65	**0.01**	−5.14 ± 0.54	0.053	−0.7 ± 0.9	0.35
	Male	33	0.35 ± 0.8		−0.21 ± 0.67		−2.87 ± 0.8		−15 ± 0.52		−7.14 ± 0.67		−0.56 ± 0.97	
Disease's duration	<1 year	22	−0.31 ± 0.99	0.82	−1.01 ± 1.06	0.82	−2.7 ± 1.01	0.71	−13.8 ± 0.67	0.5	−5.9 ± 0.57	0.55	0.37 ± 0.8	0.68
	1 year	10	−0.96 ± 2.1		−1.44 ± 1.6		−4.08 ± 1.5		−15.2 ± 1.01		−8.65 ± 1.9		−3.5 ± 2.6	
	≥2 year	12	0.15 ± 0.65		−0.31 ± 0.62		−2.6 ± 0.6		−14.7 ± 0.72		−6.15 ± 0.38		−00.6 ± 0.65	
Tumour size (cm)	<500 mm^2^	14	0.48 ± 1.04	0.99	−0.01 ± 0.99	0.74	−2.17 ± 1.06	0.68	−13.8 ± 0.82	0.055	−6.3 ± 0.51	0.21	0.24 ± 1.01	0.77
	500–800 mm^2^	15	0.29 ± 0.95		−0.13 ± 0.79		−2.12 ± 0.78		−13.5 ± 0.6		−5.6 ± 0.77		0.42 ± 0.85	
	>800 mm^2^	15	−1.7 ± 1.5		−2.5 ± 1.4		−4.6 ± 1.28		−15.7 ± 0.81		−7.9 ± 1.25		−2.47 ± 1.8	
CSF leak	No	20	0.75 ± 0.74	0.5	−0.04 ± 0.67	0.71	−2.8 ± 0.99	0.48	−14.16 ± 0.6	0.47	−6.16 ± 0.77	0.2	−0.49 ± 1.03	0.51
	Low flow	10	−0.59 ± 1.03		−0.93 ± 1.06		−3.23 ± 1.11		−15.3 ± 0.59		−7.14 ± 0.55		−0.73 ± 1.15	
	High flow	14	−1.69 ± 1.77		−2.15 ± 1.65		−2.98 ± 1.17		−14.1 ± 1		−6.96 ± 1.25		−0.7 ± 1.76	
Knosp classification	1	10	0.3 ± 0.88	0.87	−0.2 ± 0.74	0.73	−2.4 ± 0.65	0.66	−14.3 ± 0.54	0.64	−5.37 ± 0.46	0.24	0.079 ± 0.8	0.87
	2	16	−0.09 ± 0.88		−0.52 ± 0.83		−2.62 ± 0.84		−14.1 ± 0.64		−6.2 ± 0.73		0.87±−0.11	
	3	18	−0.89 ± 1.47		−1.67 ± 1.37		−3.64 ± 1.29		−14.7 ± 0.91		−7.73 ± 1.08		−1.4 ± 1.65	
Invasiveness	Invasive	7	0.14 ± 0.74	0.08	−0.3 ± 0.61	0.056	−2.84 ± 0.72	0.22	−14.3 ± 0.46	0.64	−6.7 ± 0.63	0.78	−0.58 ± 0.87	0.45
	Non invasive	37	−2.83 ± 1.81		−4.18 ± 2.2		−3.8 ± 0.8		−14.7 ± 1.48		−5.9 ± 0.55		−0.81 ± 1.3	
Drug history	Yes	9	−0.29 ± 0.74	0.91	−0.79 ± 0.6	0.96	−3.43 ± 0.7	0.42	−14.8 ± 0.45	0.089	−6.91 ± 0.61	0.3	−1.12 ± 0.89	0.44
	No	35	−0.48 ± 1.95		−1.4 ± 2.2		−1.26 ± 1.26		−12.6 ± 1.22		−5.56 ± 1		1.34 ± 1.22	

*Note*: Mann–Whitney test and Kruskal–Wallis one‐way anova were used for comparing gene expression levels between different subgroups.

## 4 DISCUSSION

NFPAs are a group of histologically benign tumours that are associated with morbidity and mortality.^3^ Therefore, identification of the molecular events leading to the development of NFPAs is important. In the current experimental study, we have selected six lncRNAs, namely MAPKAPK5‐AS1, NUTM2B‐AS1, ST7‐AS1, LIFR‐AS1, PXN‐AS1 and URB1‐AS1 to assess their expression in a cohort of Iranian patients with NFPA. Expression assays showed up‐regulation of MAPKAPK5‐AS1, PXN‐AS1 and URB1‐AS1 in NFPA tissues compared with control samples. Previous studies have shown the role of these lncRNAs in other disorders. For instance, MAPKAPK5‐AS1 have been found to promote progression of colorectal cancer through regulation of expression of MK5 and acting as a sponge for let‐7f‐1‐3p.^11^ PXN‐AS1‐L has a role in progression of nasopharyngeal carcinoma through upregulation of SAPCD2.^12^ Moreover, it enhances progression of non‐small cell lung cancer via regulation of PXN.^13^ However, the role of these lncRNAs in PAs have not been investigated.

The depicted ROC curves showed the AUC values of 0.73, 0.80 and 0.73 for MAPKAPK5‐AS1, PXN‐AS1 and URB1‐AS1, respectively. Therefore, these lncRNAs represent potential markers for diagnostic approaches in PAs.

Moreover, we discovered significant correlations between expression levels of MAPKAPK5‐AS1, NUTM2B‐AS1, ST7‐AS1, LIFR‐AS1, PXN‐AS1 and URB1‐AS1 in PAs and control samples. These correlations might indicate functional links between these lncRNAs.

Relative expression level of PXN‐AS1 was associated with tumour subtype. Therefore, this lncRNA might specifically affect pathogenesis of a certain subtype of PA or might have a more fundamental effect in the proliferation of certain types of cells. Besides, relative expression levels of MAPKAPK5‐AS1 and LIFR‐AS1 were associated with gender of patients. This observation may reflect functional relation between these lncRNAs and sex hormones or hormone responsive elements. Future studies are needed to elaborate the underlying mechanism of these observations. Cumulatively, the current study indicates the possible role of MAPKAPK5‐AS1, PXN‐AS1 and URB1‐AS1 lncRNAs in the pathogenesis of NFPAs. These results should be verified by functional studies and replicate studies in larger cohorts of patients.

## AUTHOR CONTRIBUTIONS

**Mohammad Taheri:** Data curation (equal); supervision (equal). **Arash Safarzadeh:** Investigation (equal); methodology (equal). **Solat Eslami:** Formal analysis (equal); visualization (equal). **Nader Akbari Dilmaghani:** Investigation (equal); validation (equal). **Soudeh Ghafouri‐Fard:** Supervision (equal); validation (equal); writing – original draft (equal). **Guive Sharifi:** Investigation (equal); project administration (equal). **Arefe Bahranian:** Investigation (equal); methodology (equal).

## CONFLICT OF INTEREST STATEMENT

The authors declare they have no conflict of interest.

## Supporting information


Table S1.

## Data Availability

The analysed datasets generated during the study are available from the corresponding author upon reasonable request.
