# Supplementary material for: Upregulation of MAPKAPK5‐AS1, PXN‐AS1 and URB1‐AS1 lncRNAs in non‐functioning pituitary adenoma
Source: J Cell Mol Med. 2023 May 8;27(11):1550–6. doi: 10.1111/jcmm.17763 (PMC10243150; doi:10.1111/jcmm.17763)
Supplement: Supplementary file 1 — Table S1. [file JCMM-27-1550-s001.docx]

Table S1. Details of demographic data (CD: cystic degeneration, AP: apoplexy).

| Sample number | Diagnosis (subtypes) | Gender | age  (year) | disease duration (m/d/y) | Tumor Size (mm^2^) | first operation/re-operation | macro/micro/giant | CSF leak | Knosp classification | hardy classification | invasiveness | treatment  strategy | Drug history |
| --- | --- | --- | --- | --- | --- | --- | --- | --- | --- | --- | --- | --- | --- |
| 1 | NFPA+CD | M | 38 | 2y | 900 | first | macro | low flow | 3a | 3 | noninvasive | surgery | - |
| 2 | NFPA | M | 67 | 2m | 125 | first | macro | low flow | 3a | 3 | non invasive | surgery | losartan+metformin |
| 3 | NFPA | M | 39 | 2y | 750 | re op | macro | High flow | 3a | 3 | non invasive | surgery | - |
| 4 | NFPA+AP+CD | F | 28 | 1y | 400 | first | macro | no | 1 | 3 | non invasive | surgery | - |
| 5 | NFPA | F | 46 | 6 m | 588 | first | macro | high flow | 1 | 3 | non invasive | surgery | - |
| 6 | NFPA+CD | M | 44 | 2w | 980 | first | macro | low flow | 2 | 3 | non invasive | surgery | - |
| 7 | NFPA | M | 35 | 1m | 1050 | re op | macro | no | 1 | 3 | non invasive | surgery | - |
| 8 | NFPA | F | 53 | 5y | 40 | First | micro | low flow | 1 | 1 | non invasive | surgery | - |
| 9 | NFPA | M | 52 | 1y | 440 | First | macro | no | 2 | 3 | non invasive | surgery | - |
| 10 | NFPA | M | 68 | 1y | 2250 | first | giant | high flow | 2 | 4 | invasive | surgery | - |
| 11 | NFPA+AP+CD | M | 56 | 20d | 1457 | first | giant | high flow | 1 | 4 | invasive | surgery | - |
| 12 | NFPA | M | 59 | 6m | 216 | first | macro | no | 1 | 3 | non invasive | surgery | - |
| 13 | NFPA | f | 61 | 10m | 644 | first | macro | low flow | 2 | 3 | non invasive | surgery | - |
| 14 | NFPA | M | 50 | 3y | 1056 | first | macro | high flow | 3a | 3 | non invasive | surgery | - |
| 15 | NFPA | M | 42 | 1m | 1440 | first | giant | no | 3a | 3 | non invasive | surgery | - |
| 16 | NFPA | F | 52 | 2m | 621 | first | macro | no | 2 | 3 | non invasive | surgery | - |
| 17 | NFPA | M | 48 | 2m | 360 | first | macro | no | 1 | 3 | noninvasive | surgery | - |
| 18 | NFPA+AP | M | 63 | 1.5m | 675 | First | macro | no | 2 | 3 | non invasive | surgery | pantoprazole+rosuvastatin+losartan |
| 19 | NFPA+CD | M | 35 | 1y | 1050 | first | macro | high flow | 3a | 3 | non invasive | surgery | - |
| 20 | NFPA | M | 47 | 6 m | 660 | first | macro | high flow | 1 | 3 | non invasive | surgery | - |
| 21 | NFPA | M | 46 | 1y | 840 | first | macro | no | 3a | 3 | non invasive | surgery | - |
| 22 | NFPA+AP+CD | F | 38 | 1y | 270 | first | macro | no | 2 | 3 | non invasive | surgery | - |
| 23 | NFPA | M | 72 | 2y | 72 | first | micro | low flow | 2 | 1 | non invasive | surgery | - |
| 24 | NA | M | NA | NA | NA | NA | NA | NA | NA | NA | NA | NA | NA |
| 25 | NFPA | M | 57 | 3y | 168 | first | macro | low flow | 2 | 3 | non invasive | surgery | famotidine |
| 26 | NFPA+CD | M | 42 | 2y | 425 | first | macro | high flow | 2 | 3 | non invasive | surgery | - |
| 27 | NFPA+CD | M | 69 | 8m | 550 | first | macro | no | 2 | 3 | non invasive | surgery | - |
| 28 | NFPA | M | 67 | 3m | 660 | first | macro | low flow | 3a | 3 | invasive | surgery | losartan |
| 29 | NFPA | M | 68 | 2y | 500 | first | macro | no | 1 | 3 | non invasive | surgery | - |
| 30 | NFPA | M | 61 | 1y | 840 | first | giant | no | 4 | 4 | invasive | surgery | - |
| 31 | NFPA | M | 67 | 5y | 1302 | first | giant | low flow | 3b | 3 | noninvasive | surgery | - |
| 32 | NFPA | M | 64 | 8y | 400 | re op | macro | High flow | 1 | 3 | non invasive | surgery+radiotherapy(1397) | - |
| 33 | NFPA+AP | M | 43 | 10d | 1800 | first | giant | no | 3a | 3 | invasive | surgery | - |
| 34 | NFPA | F | 49 | 8m | 1470 | first | giant | High flow | 3b | 4 | invasive | surgery | losartan+metformin |
| 35 | NFPA | M | 46 | 4m | 783 | first | macro | no | 2 | 3 | non invasive | surgery | - |
| 36 | NFPA | F | 77 | 3m | 528 | first | macro | high flow | 3a | 3 | invasive | surgery | - |
| 37 | NFPA | F | 36 | 3y | 616 | first | macro | high flow | 3a | 3 | non invasive | surgery | - |
| 38 | NFPA+CD+AP | M | 49 | 40d | 378 | first | macro | low flow | 2 | 3 | non invasive | surgery | - |
| 39 | NFPA | M | 45 | 1y | 750 | first | macro | High flow | 3b | 3 | noninvasive | surgery | - |
| 40 | NFPA | M | 62 | 2w | 440 | first | macro | no | 3b | 3 | noninvasive | surgery | zimpet |
| 41 | NA | M | NA | NA | NA | NA | NA | NA | NA | NA | NA | NA | NA |
| 42 | NFPA | F | 33 | 1y | 1050 | first | macro | no | 3a*b | 3 | noninvasive | surgery | losartan,levothyroxin |
| 43 | NFPA | F | 38 | 2y | 360 | first | macro | high flow | 2 | 3 | non invasive | surgery | - |
| 44 | NFPA | M | 42 | 6M | 616 | FIRST | macro | no | 2 | 3 | non invasive | surgery | - |
| 45 | NFPA | M | 47 | 3m | 744 | first | macro | no | 2 | 3 | non invasive | surgery | methadone |
| 46 | NA | F | NA | NA | NA | NA | NA | NA | NA | NA | NA | NA | - |
| 47 | NFPA | M | 28 | 1y | 1209 | first | macro | no | 3a | 3 | non invasive | surgery | - |
